# Parents’ sports-related behaviors, self-concept of ability, interest and organized after-school sports activities participation among Chinese elementary school children

**DOI:** 10.3389/fpsyg.2025.1581296

**Published:** 2025-04-28

**Authors:** Chuantong Jiang, Norsilawati Abdul Razak, Nelfianty Mohd Rasyid, Hui Cheng

**Affiliations:** ^1^Faculty of Physical Education and Health, Zhaoqing University, Zhaoqing, China; ^2^Faculty of Sports Science and Coaching, Sultan Idris Education University, Tanjong Malim, Malaysia; ^3^School of Economics and Management, Zhaoqing University, Zhaoqing, China

**Keywords:** parents’ sports-related behaviors, self-concept of ability, interest, organized sports activities, elementary school children

## Abstract

**Background:**

Grounded in Expectancy-Value Theory, this study investigated how parents’ sports-related socialization behaviors in the family context influence their children’s participation in organized after-school sports activities. More precisely, the research analyzed the mediating effect of self-concept of ability and interest in the relationship between parents’ sports-related socialization behaviors and children’s participation in organized after-school sports activities.

**Methods:**

The research sample was derived from elementary schoolchildren in the 5th and 6th grades in urban areas of Zhaoqing, China. A total of 367 participants completed the questionnaire (177 boys; 190 girls; *M*_age_ = 11.17 years; *SD* = 0.663; age range = 10–12 years). The collected data were then utilized for further structural equation modeling (SEM) analysis.

**Results:**

The results indicated that parents’ sports-related socialization behaviors had significant direct and indirect effects on children’s participation in organized after-school sports activities. Specifically, the self-concept of ability and interest mediated the relationship between parental behaviors and children’s sports activities participation, respectively. Also, the self-concept of ability and interest serially mediated the relationship between parental behaviors and children’s sports activities participation.

**Conclusion:**

These findings underscore the crucial role parents play in their children’s participation in organized after-school sports activities. The study implies that initiatives should be taken to guide parents to perform positive family-supportive behaviors, such as offering encouragement, participating in co-activities, providing activity-related materials, and acting as role models. These behaviors can not only directly contribute to children’s participation in after-school sports activities but also indirectly influence it by enhancing children’s self-concept of sports ability and cultivating their interest in sports activities.

## 1 Introduction

Regular participation in physical activity plays a pivotal role in children’s growth and development, exerting a positive influence on their physical health, emotional well-being, cognitive abilities, and the adoption of healthy lifestyles (Carayanni et al., 2020; Kennewell et al., 2022). Although the benefits of physical activity have been confirmed by numerous studies, the phenomenon of children not meeting recommended levels of physical activity is widespread worldwide, particularly in East Asian countries such as China (Guthold et al., 2020). The World Health Organization (2022) shows that progress toward the World Health Organization (2018) goal of reducing physical inactivity by 15% by 2030 has been slow. Therefore, further research on physical activity is of great significance. Theoretically, it can contribute to a deeper understanding of the mechanisms and influencing factors of physical activity. Practically, it can provide robust support for developing targeted strategies, improving children’s physical activity levels, and advancing the goals of the global action plan.

As Liu et al. (2023) states, “organized sports and physical activity are one of the most common leisure -time activities for children and adolescents.” Organized sports usually refer to a kind of physical activity that is supervised by leaders and has corresponding rules, organized training and competitive events (Logan et al., 2019). Physical activity is generally defined as bodily movement produced by skeletal muscles that results in energy expenditure above the basal metabolic level (Caspersen et al., 1985). According to Kowalski et al. (1997), physical activity was described as “sports, games, or dance that make you breathe hard, make your legs feel tired, or make you sweat.” In elementary schoolchildren, it covers a wide range of organized and unorganized physical activities, such as school physical education, extracurricular sports club activities, and transportation physical activities such as cycling or walking as a daily means of transportation (García and Suárez, 2020). In China, to promote children’s active and healthy lifestyle, the government and relevant departments systematically encourage them to participate in extracurricular sports activities. For example, the implementation of the “double reduction” policy has effectively promoted children’s participation in organized after-school sports activities (Tan et al., 2023). Therefore, this study mainly focuses on organized after-school sports activities, namely sports activities or training courses organized by schools or institutions and conducted outside of school hours, which can be regarded as a specific form of higher-level physical activity (Nelson et al., 2011). Previous studies have shown that organized sports activities not only helps children develop an active and healthy lifestyle but also provides them with a developmental environment that fosters behavioral and psychological adjustment, enabling them to better navigate the challenges of growth (Bjørnarå et al., 2021; Jaf et al., 2021).

Moreover, organized after-school sports activities in childhood may be more susceptible to interpersonal constraints from parents than characteristics of teachers and the wider school environment (Eccles and Wigfield, 2020; Jaf et al., 2021; Wilk et al., 2018). In fact, many studies have shown that family-related variables are a particularly important social situational factor in children’s sports activities participation in decision-making, and to some extent influence individual intrinsic motivational processes related to children’s sports activities (Eccles and Wigfield, 2024; Jaf et al., 2021; Männikkö et al., 2020; Matos et al., 2021; Sutcliffe et al., 2024). More specifically, family-based studies on the impact of sports activities participation investigated family socioeconomic and cultural status, such as parental education level, family economic income (Cheung, 2017; Fernandes et al., 2012; Qunito Romani, 2020; Wilk et al., 2018), parents’ and siblings’ sports-related socialization behaviors (Reimers et al., 2019a), and intra-individual factors, such as beliefs about sports activities-related abilities (Laird et al., 2018), and perceptions of the value of activity tasks (Jaf et al., 2021). However, previous studies also had certain limitations, as well as problems that require further attention. On the one hand, several studies on children’s sports activities participation have investigated only the influence of parents’ sports-related behavioral factors (Chung and Green, 2023; Liu et al., 2017; Motoki et al., 2023; Timperio et al., 2013). On the other hand, although there are also studies that have paid attention to individual intrinsic motivation processes, a more comprehensive set of factors needs to be used to test the model and examine the interaction between them (Motoki et al., 2023).

Overall, children’s sports activities participation exhibits a complexity of external and internal patterns, especially during the transitional period of adolescence, not only focusing on the ongoing impact of parental behavior on children’s sports activities participation, but also further considering the role of individual intrinsic motivational processes (Li and Moosbrugger, 2021). Relevant theories and research advancements fully demonstrate that parental behaviors, viewed as content-specific parenting practices (Darling and Steinberg, 1993), may have a considerable impact on children’s self-perceptions of competence, value beliefs, and participation in organized after-school sports activities (Eccles and Wigfield, 2024). Therefore, based on Eccles and Wigfield (2020) parental motivational socialization model, this study examines the relationship between parents’ sports-related socialization behaviors and children’s participation in organized after-school sports activities, as well as the underlying mechanisms between them.

### 1.1 Parents’ sports-related socialization behaviors as predictors of children’s sports activities

Based on Eccles and Wigfield (2024), parental sports-related socialization has a direct impact on children’s continued participation in or withdrawal from sports activities. Specifically, parental behavior in sports activities can influence children’s sports activities participation through a variety of positive supportive behaviors, such as encouragement, co-activity, provision of activity-related materials, and role-modeling (Reimers et al., 2019a; Reimers et al., 2019b; Simpkins et al., 2012, 2015; Timperio et al., 2013). Similar findings were reported by Liszewska et al. (2018), who demonstrated that parental collaborative social control, encouragement of sports activities, positive social control, and role modeling can positively predict children’s participation in sports activities. Notably, these associations are consistent across different genders, residential areas, and school grades (Liu et al., 2017; Simpkins et al., 2012).

Furthermore, researchers have emphasized different aspects of parental behaviors in their studies on the influence of parents on children’s participation in organized after-school sports activities. For example, several studies have primarily focused on parental role-modeling (Carayanni et al., 2020; Wilk et al., 2018). A recent study highlighted the predictive role of parental encouragement and co-activity on children’s sports activities (Jaf et al., 2023). Other studies have provided evidence that parental role modeling, encouragement, and co-activity can predict children’s sports activities participation (Babkes and Weiss, 1999; Davison et al., 2003; Green and Chalip, 1997; Liszewska et al., 2018). Additionally, some research has treated these parental behaviors as an overall construct to investigate the relationship between parental support and children’s sports participation (Heitzler et al., 2010; Kwon et al., 2016; Liszewska et al., 2018; Wilk et al., 2018). However, few studies have incorporated the provision of materials (e.g., sports equipment and transportation) into models of parental influence (Simpkins et al., 2015). In this study, parental behaviors are considered across these four dimensions. Overall, these findings underscore the positive association between parents’ sports-related socialization behaviors and children’s sports activities participation. Thus, the research hypothesis proposed is as follows:

*Hypothesis* 1: Parents' sports-related socialization behaviors are positively associated with children's participation in organized after-school sports activities.

### 1.2 Children’s self-concept of ability and interest as the underlying mechanisms

According to Eccles and colleagues’ model of parents’ socialization of motivation, parental behavior indirectly determines children’s participation in specific activities by influencing their self-concepts of ability and interest (Eccles and Wigfield, 2024). In the domain of sports activities, the self-concept of ability refers to children’s internal perception and evaluation of their competence in sports activities, while interest represents their intrinsic subjective value judgment toward sports activities (Eccles et al., 2005). Previous research has demonstrated positive associations between parental behaviors and children’s self-perceptions of sports competence and value beliefs. For instance, Heitzler et al. (2010) found that parental social support predicts children’s self-efficacy in sports activities. Moreover, Babkes and Weiss (1999) showed that parents’ positive role modeling in exercise fosters more favorable beliefs in children’s abilities. These findings are in line with Bandura’s self-efficacy theory which also emphasizes the role of vicarious experiences in shaping self-perception (Bandura, 1997). Specifically, when children observe their parents or other role models successfully engaging in sports activities, it can enhance their belief that they themselves are also capable of doing the same. In addition, Jaf et al. (2023) revealed that children whose parents attend their training sessions and competitions perceive sports activities as enjoyable, important, and useful. Furthermore, extensive research has indicated that children’s perceptions of competence and value in sports activities positively predict their participation in such activities (Pelletier et al., 2020; Simpkins et al., 2012).

Moreover, several studies have examined the mediating role of the self-concept of ability in the relationship between parental behaviors and children’s participation in sports activities. For example, Laird et al. (2018) demonstrated that social support may enhance girls’ self-efficacy, leading them to view their abilities in sports activities more positively, thereby increasing their motivation to participate and their determination to overcome challenges, ultimately influencing their actual sports activities levels. Additionally, Timperio et al. (2013) found that parents’ sports activities behaviors not only serve as role models but also indirectly promote children’s participation by shaping their perceptions of their own physical competence. Similarly, Jaf et al. (2021) found that sports-related family co-activities significantly predict children’s anticipated sports-related behaviors through their sports value beliefs. Furthermore, Pelletier et al. (2020) demonstrated that children’s perceptions of competence and value mediate the relationship between parental support and children’s participation in sports activities. Simpkins et al. (2012) also reported consistent findings, showing that parental behaviors indirectly influence children’s sports activities participation through their perceptions of competence and value, with these relationships unaffected by gender differences. Overall, based on the evidence above, it can be concluded that children’s self-concept of ability and value beliefs in sports activities mediate the relationship between parental behaviors and children’s sports activities participation. Therefore, research hypotheses 2 and 3 are proposed as follows:

*Hypotheses:* Self-concept of ability (*H2*) and interest (*H3*) respectively mediate the relationship between parents' sports-related socialization behaviors and children's participation in organized after-school sports activities.

It is worth noting that children’s self-concept of ability may affect their interest in participating in specific activities (Viljaranta et al., 2014; Wu et al., 2020). According to Eccles et al. (1983) and Eccles and Wigfield (2024), students’ self-concept of ability lays the foundation for their interest in specific subjects, which can be analogously applied to the domain of sports activities. That is, children’s self-concept of ability in sports influences their interest in sports activities, which in turn is linked to their participation levels. For example, those who excel in soccer are often more willing to participate in school soccer matches or extracurricular soccer activities. This is because individuals tend to engage in activities they believe they can perform well and derive a sense of achievement from, and sports activities are no exception. A positive self-concept of ability fosters high self-efficacy, which stimulates children’s enthusiasm for exploring and participating in sports activities, thereby enhancing their interest in those activities (Munroe-Chandler et al., 2008). This interest, in turn, further motivates them to engage more actively in sports activities (Van Yperen et al., 2022). In summary, based on the aforementioned theories and related research, it can be inferred that parents’ sports-related socialization behaviors may predict children’s participation in organized after-school sports activities through a sequential pathway involving their self-concept of ability and interest in sports activities. Therefore, the research hypothesis proposed is as follows:

*Hypothesis* 4: Self-concept of ability and interest serially mediate the relationship between parents' sports-related socialization behaviors and children's participation in organized after-school sports activities.

### 1.3 Child gender

Parental socialization factors are positively correlated with their children’s sports beliefs and participation levels, while gender stereotypes often exist in parents’ beliefs and practices (Eccles et al., 1983; Eccles and Wigfield, 2024; Fredricks and Eccles, 2005; Simpkins et al., 2012). Previous studies on the influence of gender focused on the differences in the average levels of related concepts. For example, Simpkins et al. (2012) found that children of different genders have different perceptions of their abilities and value of different tasks, but there are no significant gender differences in their behavioral performances. Moreover, parents’ material provisions and encouragement often differ by children’s gender, but there are no significant differences in parental role modeling and co-activities with children (Simpkins et al., 2012). However, Jaf et al. (2021) reported inconsistent findings, except for parental role modeling. In the sample of Chinese children, Wang et al. (2017) found that boys engage in more moderate to vigorous physical activity than girls, but no gender differences in parental logistical support, explicit modelling, and their physical activity. Hong et al. (2020) also reported similar findings, except for gender differences in parental support. As a result, these previous studies indicate that the influence of gender factors should be fully considered in the research process.

Furthermore, Simpkins et al. (2012) found that boys were stronger than girls in the relationship between adolescent beliefs and their behavior. Conversely, Jaf et al. (2021) showed that gender did not have a significant moderating effect in the relationship between family socialization behavior, adolescent sports value beliefs, and their sports participation behavior. However, there has been a limited number of studies investigating gender as a moderating factor between parental sports-related behavior, children’s motivation and participation in sports activities. As Simpkins et al. (2012) stated, most previous studies have shown that the relationship between parents’ socialization behaviors and children’s beliefs and behaviors in sports activities is not influenced by gender. As a consequence, we expected that the parental socialization model proposed by Eccles and Wigfield (2024) would be equally applicable to both the boys and girls in our sample. Thus, the following hypothesis is proposed:

*Hypothesis* 5: The relations between parents' sports-related behaviors, children's self-concept of ability, interests, and their participation in organized after-school sports activities are invariant across child gender.

### 1.4 The present study

**Figure 1:**
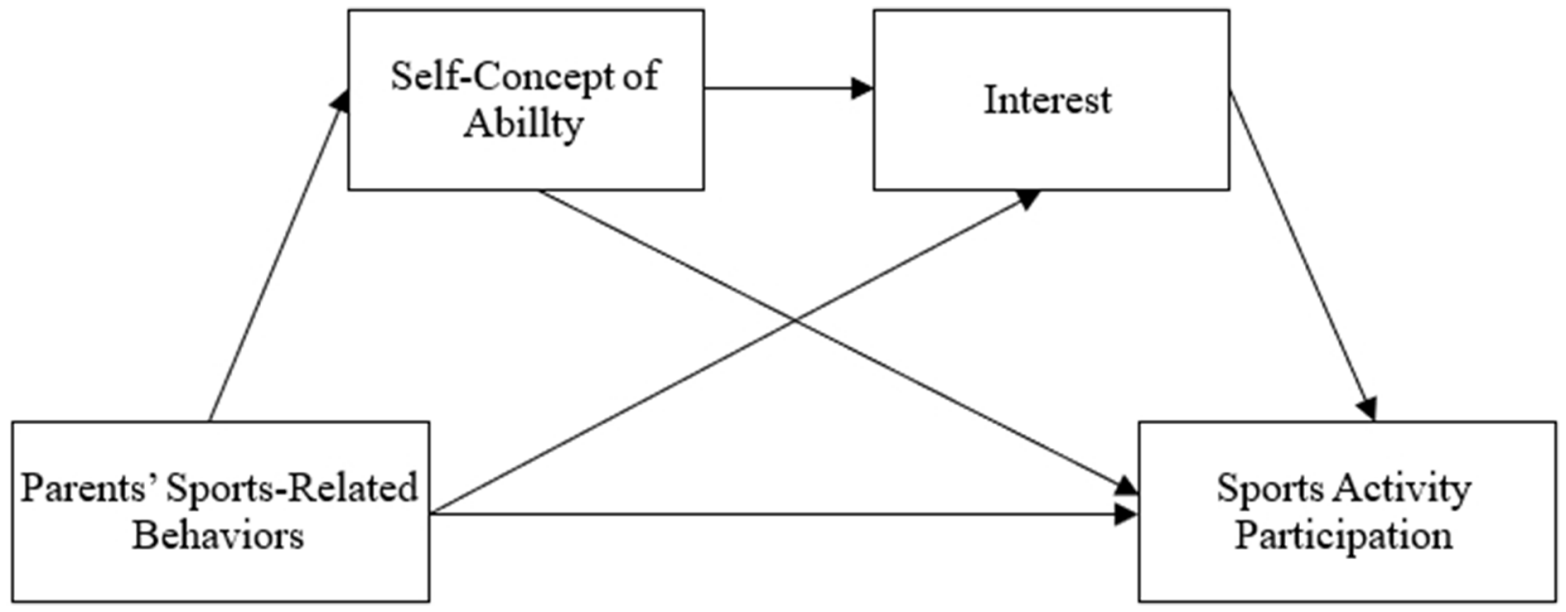
Proposed serial mediation model.

The promotion of children’s sports activities is a global topic, and within this context, the crucial role of family influence in the development of children’s sports is receiving growing attention. However, little research has comprehensively integrated and investigated the mechanism of parental sports-related socialization behavior on children’s participation in organized after-school sports activities in the context of the Chinese elementary schoolchildren demographic. Therefore, based on relevant theories and research advancements, the current study proposes a multivariate mediation model (see Figure 1) to investigate the relationship between parents’ sports-related socialization behaviors and children’s sports activities participation, as well as potential mediation mechanisms. Specifically, the present study aims (1) to comprehensively explore the impact of parents’ sports-related socialization behaviors on children’s organized after-school sports activities participation, (2) to investigate the mediating role of self-concept of ability or interest in the relationship between parents’ sports-related socialization behaviors and children’s organized after-school sports activities, and (3) to identify the serial mediating role of self-concept of ability and interest in the connection between parents’ sports-related socialization behaviors and children’s organized after-school sports activities. The research findings can provide valuable insights for promoting physical activities among Chinese children and contribute to the development of their sports and the cultivation of healthy lifestyles.

## 2 Materials and methods

### 2.1 Sample and procedure

Participants in this study were selected from elementary schoolchildren (grades 5 and 6) aged between 10 and 12 in the urban area of Zhaoqing, China. The sample size was determined using Jackson (2003) *N:q* rule, which recommends a ratio of 10:1 between the sample size (*N*) and the number of parameters to be estimated (*q*; Kline, 2015, p. 16). In this study, the number of parameters to be estimated was 34, necessitating a minimum sample size of 340. Considering Salkind (1997) recommendation for oversampling, an additional 10% was added, resulting in a final sample size of 374. To ensure the randomness and representativeness of the sample, a stratified random cluster sampling method was employed. By stratification according to grade and taking class as sampling unit, 8 classes from 7 schools were randomly selected, with 4 classes in each grade. The questionnaires were translated into Chinese using the back-translation method (Brislin, 1970). Subsequently, upon obtaining approval from the education administration and schools, as well as informed consent from students and parents, the survey began to be implemented. Investigators who had received research ethics training distributed and collected the questionnaires in the selected classes, and a total of 367 valid questionnaires were collected, which met the sample size requirements for structural equation modeling (SEM) analysis (Hair et al., 2018). All recruitment and data collection procedures were approved by the Science and Technology Ethics Committee of Zhaoqing University.

### 2.2 Measures

**Parents’ sports-related socialization behaviors**. A five-item questionnaire measured four dimensions of parents’ sports-related positive behavior. For example, one item was used to ask on how often their parents engaged in physical activities after work or at weekends (i.e., role-modeling): “How often do your parents engage in sports or physical activities?” on a 5-point scale ranging from 1 (never) to 5 (every day). One item was used to assess parents’ encouragement: “How much do your parents encourage you to participate in sports or physical activities? (1 = strongly discourage, 5 = strongly encourage).” One item was used to assess parents’ provision of activity-related materials: “Did your parents provide logistic support (i.e., provision of equipment, books or transportation, etc.) for you in the last year?” on a 5-point scale ranging from 1 (never) to 5 (always). Two items were used to assess parents’ sports-related co-activities: “How often do your parents participate in sports or physical activities with you? (1 = never, 5 = almost every day for a long while),” “How often do your parents take you to paid sporting events? (1 = never, 5 = weekly).” The higher the measurement score for these items indicates the greater the child’s perceived support from their parents. These items follow theoretical guidelines (Eccles et al., 1983; Fredricks and Eccles, 2005), as well as other scholars’ use of similar items to measure parental sports-related behavior (Jaf et al., 2021; Liu et al., 2017; Simpkins et al., 2012). In this study, the questionnaire demonstrated a excellent level of internal consistency (*α* = 0.93).

**Self-Concept of Abillty**. The scale of children’s self-concept of abillty, originally developed and validated by Eccles et al. (1983, 2005) and recently applied in sports by Simpkins et al. (2015), was used to assess children’s perceptions of ability in sports activities. The scale consists of 4 items, including (1) “How good at sports are you? (1 = not very good, 5 = very good),” (2) “If you were to list all the students from best to worst in sports where are you? (1 = one of the worst, 5 = one of the best),” (3) “Compared to other subjects how good are you at sports? (1 = a lot worse, 5 = a lot better),” (4) “How good would you be at learning something new in sports? (1 = not very good, 5 = very good).” Similar items have been used in previous studies (Fredricks and Eccles, 2005) and have been shown to have high internal consistency and predictive validity (*α* = 0.81–0.89). In this study, the scale demonstrated a excellent level of internal consistency (α = 0.91).

**Interest**. Children’s interest in sports activities was measured using the Subjective Task Value Scale (Eccles et al., 1983, 2005; Fredricks and Eccles, 2005). Based on factor analysis and theoretical considerations, this study only used the interest part of the scale. This specific scale measures intrinsic value components, with three items measuring how much children enjoy after-school sports activities, including “In general, I find playing sports? (1 = very boring, 5 = very interesting),” “How much do you like doing playing sports? (1 = a little, 5 = a lot),” “Compared to most of your other activities, how much do you like sports? (1 = not as much, 5 = a lot).” In the evaluation of these items, the higher the score, the greater the children’s interest in organized after-school sports activities. This scale has been shown to have good reliability in a series of previous studies (α = 0.81–0. 92). In this study, the scale demonstrated a good level of internal consistency (α = 0.85).

**Organized Sports Activities**. To measure children’s participation in organized after-school sports activities, this study used similar items used in the analysis by Fredricks and Eccles (2005) and Simpkins et al. (2015). The questionnaire consisted of two items, including “How often do you play sports on organized teams where someone keeps score?” and “How much time do you spend on taking part in organized sports every week?.” Both items used a 5-point Likert scale (1 = never, 2 = almost never, 3 = less than once per week, 4 = a couple times per week, 5 = almost every day). Higher item scores indicate higher levels of participation in organized after-school sports activities. Item reliability has also been confirmed in previous studies, and in this study it also showed an acceptable level of internal consistency (α = 0.75).

### 2.3 Data analysis

This study used SPSS 25 and AMOS 24 software to conduct data analysis, including descriptive statistics and inferential statistics. In the preliminary data analysis stage, the collected data were first screened, including missing value interpolation, outlier detection, and normality assessment. Descriptive statistics were carried out for demographic variables and related constructs, including means, standard deviations, and other key indicators, to provide a clear overview of the central tendencies and dispersion characteristics of the variables. Inferential statistics employed SEM analysis to achieve the objectives of this study. Meanwhile, correlation analysis was performed to examine the linear relationships and directions among variables, laying a solid foundation for further analysis.

In SEM analysis, the measurement model and structural model were analyzed in order according to the two-step method proposed by Anderson and Gerbing (1988). The maximum likelihood method was used to estimate the parameters of the measurement model and the structural model, and a set of multivariate indicators was used to evaluate the model’s goodness of fit (Hair et al., 2018; Jackson et al., 2009; Kline, 2015), such as normed chi-square (χ^2^/df), comparative fit index (CFI), Tucker-Lewis index (TLI), root mean square error of approximation (RMSEA), and standardized root mean square residual (SRMR). In terms of effect testing, considering that the product of the non-standardized path coefficients of the intermediary variables does not conform to the normal distribution assumption, the intermediary effect is evaluated by bootstrap method (MacKinnon, 2008; Preacher and Hayes, 2008), including re-estimating the standard error and confidence interval of the indirect effect, and then calculating the significance level of the indirect effect, according to the standard error and the unstandardized coefficient.

## 3 Results

### 3.1 Common method bias, descriptive statistics and correlation analysis

Prior to evaluating the common method bias, the data obtained were screened. Specifically, all 14 observed variables have missing data but the missing rate is less than 1%. In view of their random and irregular characteristics, we interpolated them with the Bayesian method built into the Amos software according to Buhi et al. (2008). In terms of outliers, through the analysis of histograms, box plots and the frequency distributions of standardized z-scores, it was found that the absolute values of z-scores of all items were within 4, confirming the absence of univariate outliers, and after the Mahalanobis *D^2^* measurement, it was further confirmed that there were no multivariate outliers (Hair et al., 2018). Moreover, data skewness values ranged from −1.679 to −0.704, and kurtosis values ranged from −0.695 to 2.978, within the acceptable range for univariate normality (Byrne, 2010; Hair et al., 2018). As a result, all 367 cases were retained to the next stage of data analysis, including 177 boys and 190 girls, with an average age of 11.17 years (*SD* = 0.663). Power analysis using the *R* program showed that the sample size of 367 in this study had a statistical power of 0.989, indicating high confidence in result accuracy and that the obtained data met the minimum sample size requirements (Kline, 2015, p. 290; MacCallum et al., 1996). Furthermore, the technique of single-factor confirmatory factor analysis (CFA) was employed to assess the impact of common method bias. The results of single-factor CFA were, χ^2^ (77) = 1501.557, *p* = 0.000, χ^2^/df = 19.501, CFI = 0.594, TLI = 0.520, RMSEA = 0.225, SRMR = 0.180, indicating that the data did not fit well. Moreover, the results of multi-factor CFA were, χ^2^ (71) = 93.284, *p* = 0.039, χ^2^/df = 1.314, CFI = 0.994, TLI = 0.992, RMSEA = 0.029, SRMR = 0.031. There was a significant difference between the two models (Δ*χ*^2^ = 1408.273, Δ*df* = 6, *p* < 0.001), indicating that no common method variance in the constructs of this study (Podsakoff et al., 2003). Thus, the coefficient estimates in this study would not be biased.

Then, descriptive statistics and bivariate correlation analysis were carried out. As shown in Table 1, the children’s perceived mean of parental sports-related socialization behavior was 3.77 (*SD* = 1.03), the mean of self-concept of ability was 3.55 (*SD* = 1.14), the mean of interest was 4.01 (*SD* = 0.92), and the mean of sports activities participation was 4.15 (*SD* = 0.81). These results showed that participants reported high parental modeling and support, along with strong self-ability beliefs, high interests, and active sports activities participation. Moreover, the results of the chi-square test and independent samples t-test showed that there were no significant differences between boys and girls in terms of grade, age, parental role modeling, encouragement, co-activities, self-concept of ability, and participation in sports activities, while significant differences were found in parental material provisions and children’s interests. In addition, correlation analysis showed that all bivariate estimates were statistically significant (*p* < 0.01), including parental sports-related socialization behavior and children’s sports activities participation (*r* = 0.653), and interest and sports activities participation (*r* = 0.573) showed a strong correlation, parental sports-related socialization behavior and children’s sports activities interest (*r* = 0.492), and self-concept of ability and sports activities participation (*r* = 0.381) showed a moderate correlation, and self-concept of ability showed a weak correlation with parental behavior and interest (*r* = 0.273), respectively (see Table 2). Overall, it can be seen from these results that there were no uncorrelated variables and no multicollinearity problems (Grewal et al., 2004).

**Table 1:** Results of the descriptive analysis of variables by gender (*N* = 367).

Variable	Boys	Girls	Total	t/χ^2^	*p*-value
	*n* = 177	*n* = 190	*n* = 367		
Grade, n (%)					
Grade 5	86 (48.6)	98 (51.6)	184 (50.1)	0.328	0.567
Grade 6	91 (51.4)	92 (48.4)	183 (49.9)		
Age, n (%)					
10	28 (15.8)	26 (13.7)	54 (14.7)	0.515	0.773
11	91 (51.4)	104 (54.7)	195 (53.1)		
12	58 (32.8)	60 (31.6)	118 (32.2)		
Parents behavior, mean (SD)	3.86 (0.95)	3.68 (1.09)	3.77 (1.03)	1.596	0.111
Role-modeling	3.94 (1.07)	3.81 (1.20)	3.87 (1.14)	1.070	0.285
Encouragement	3.85 (1.09)	3.70 (1.26)	3.77 (1.78)	1.251	0.212
Provision of materials	3.93 (1.10)	3.59 (1.18)	3.75 (1.15)	2.789	0.006
Co-activities	3.79 (1.02)	3.67 (1.25)	3.72 (1.08)	1.064	0.288
Self-concept of ability, mean (SD)	3.60 (1.16)	3.51 (1.12)	3.55 (1.14)	0.684	0.494
Interest, mean (SD)	4.17 (0.81)	3.85 (0.99)	4.01 (0.92)	3.362	0.001
Sports activity, mean (SD)	4.19 (0.74)	4.11 (0.86)	4.15 (0.81)	0.970	0.333

**Table 2:** Results of correlations, normality, reliability, and discriminant validity (*N* = 367).

Variable	Skewness	Kurtosis	Cronbach’s α	1	2	3	4
1. Sports activity	−1.679	2.978	0.746	**0.770**			
2. Interest	−1.311	1.062	0.854	0.573**	**0.820**		
3. Self-concept of ability	−0.704	−0.695	0.911	0.381**	0.273**	**0.850**	
4. Parents behavior	−1.199	0.311	0.930	0.653**	0.492**	0.273**	**0.870**

A diagonal element in bold represents the square root of AVE; the elements below the diagonal in the matrix are the Pearson correlation coefficients between the latent constructs; **p < 0.01.

### 3.2 Structural equation modeling analysis

A CFA evaluation was conducted for both each single measurement model and the overall measurement model prior to the structural model analysis (Anderson and Gerbing, 1988). As shown in Table 3, the results indicate that the standardized factor loadings for all measurement models met the threshold of 0.7 (*p* < 0.001). Consequently, all items were retained in the analysis (Hair et al., 2018). Moreover, the overall measurement model yielded a good fit, χ^2^(367) = 93.284, *p* = 0.039, χ^2^/df = 1.314, CFI = 0.994, TLI = 0.992, RMSEA = 0.029, SRMR = 0.031. Although the *p*-values here is statistically significant, this is likely attributable to the relatively large sample size employed in this study (Marsh et al., 2004). Furthermore, the average variance extracted (AVE) estimates and item reliability (SMC) values both exceeded the 0.5 standard, and the composite reliability (CR) values exceeded the 0.7 standard. Taken together, these results fully validate the convergent validity of the measurement model. As shown in Table 2, the arithmetic square root of the AVE of each variable was greater than the absolute value of its correlation coefficient with other variables, indicating that there are differences and discriminative validity among the variables (Fornell and Larcker, 1981).

**Table 3:** CFA results for the measurement model.

Variable	Item	Parameter significance estimation				Convergent validity			
		Unstd.	S.E.	*t*-value	*p*-value	Std.	SMC	CR	AVE
Parents behavior	PB1	1.000				0.870	0.757	0.939	0.754
	PB2	0.999	0.047	21.312	***	0.840	0.706		
	PB3	1.027	0.044	23.453	***	0.883	0.780		
	PB4	1.041	0.044	23.717	***	0.888	0.789		
	PB5	0.960	0.043	22.197	***	0.859	0.738		
Ability	SCA1	1.000				0.838	0.702	0.911	0.719
	SCA2	0.960	0.048	20.125	***	0.865	0.748		
	SCA3	0.953	0.048	19.783	***	0.855	0.731		
	SCA4	0.948	0.050	19.065	***	0.834	0.696		
Interest	IN1	1.000				0.895	0.801	0.861	0.676
	IN2	0.857	0.056	15.303	***	0.717	0.514		
	IN3	0.947	0.051	18.466	***	0.845	0.714		
Sports activity	PA1	1.000				0.798	0.637	0.747	0.597
	PA2	0.971	0.083	11.684	***	0.746	0.557		

*N* = 367. Unstd, unstandardized factor loading; S.E., standard error; *t*-value, critical ratios; ***p < 0.001; Std, standardized factor loading; SMC, item reliability; CR, composite reliability; AVE, average variance extracted.

**Figure 2:**
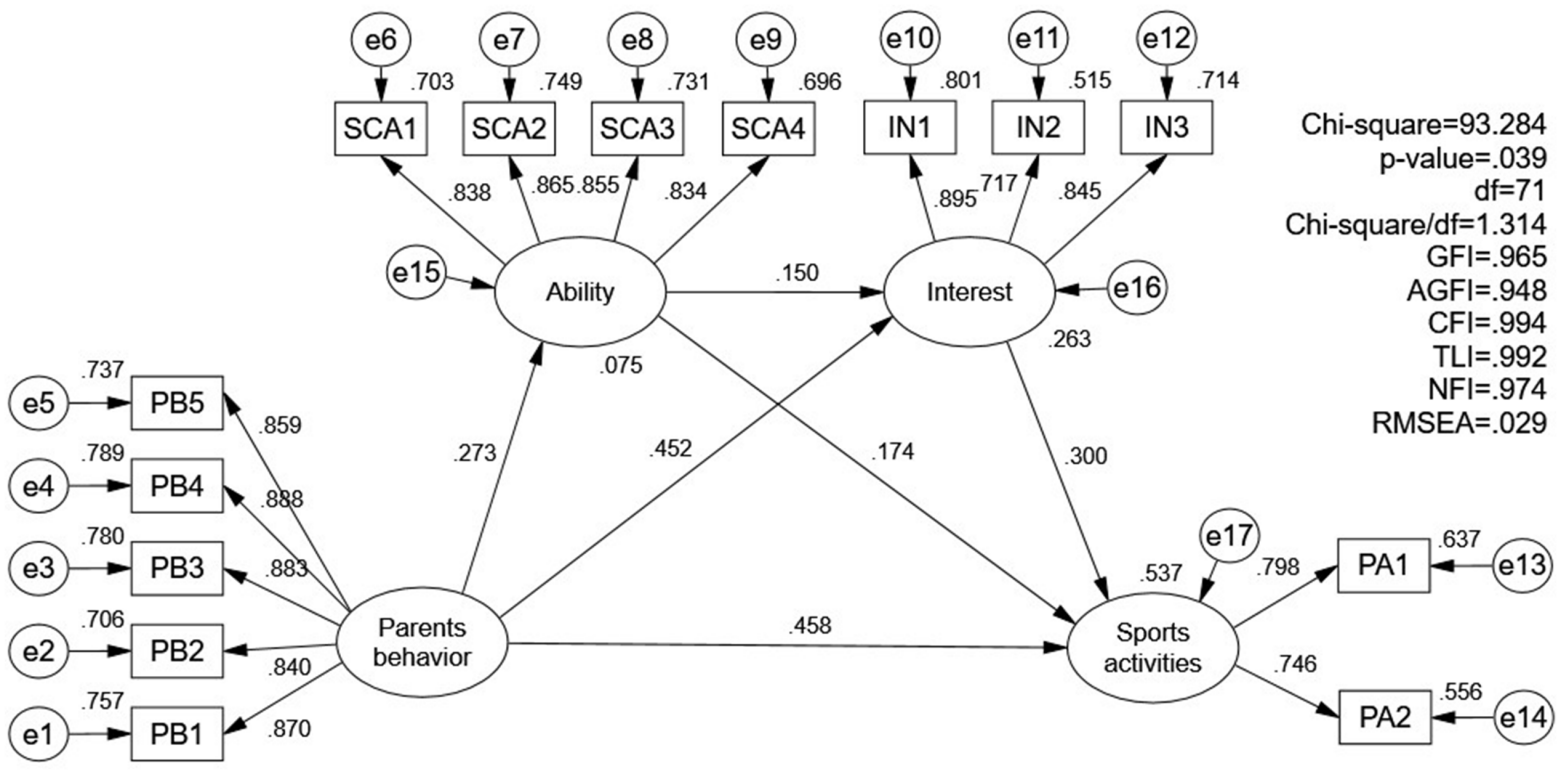
Results of structural model analysis with standardized coefficients and *R*^2^.

Next, the path coefficients between the variables and the proportion of variance explained by exogenous variables were tested through structural model analysis (see Figure 2). The results showed that children’s perceived parents’ sports-related socialization behaviors had a positive direct effect on their self-concept of ability (*β* = 0.273, *p* < 0.001), interest (*β* = 0.452, *p* < 0.001) and after-school organized sports activities (*β* = 0.458, *p* < 0.001), respectively. In turn, self-concept of ability (*β* = 0.174, *p* = 0.001) and interest (*β* = 0.300, *p* < 0.001) had a positive direct effect on children’s organized sports activities after school. Meanwhile, children’s self-conceptions of ability had a positive direct effect on their interest in participating in sports activities (*β* = 0.150, *p* = 0.006). Furthermore, the squared multiple correlation (*R*^2^) of children’s participation in after-school organized sports activities was 0.537. This figure suggests that parents’ sports-related behaviors, children’s ability beliefs, and interests together explained 53.7% of the variance in after-school sports activities. Evidently, the model exhibits substantial statistical power in explaining children’s involvement in after-school organized sports activities.

### 3.3 Mediation analyses

To address the research objectives and validate the research hypotheses, this study assessed the direct and indirect effects of parents’ sports-related socialization behaviors on children’s participation in after-school organized sports activities. The results indicated that, as a partial mediation model, parents’ sports-related behaviors had significant direct (*β* = 0.326, *Z* = 6.792, *p* < 0.001) and indirect (*β* = 0.139, *Z* = 3.971, *p* < 0.001) effects on children’s after-school sports activities (*H1*). Moreover, specific indirect effects showed that parents’ sports-related behaviors positively predicted their participation in after-school sports activities through children’s self-concept of ability (*β* = 0.034, *Z* = 2.429, *p* = 0.007) and interest (*β* = 0.097, *Z* = 3.593, *p* < 0.001), which supported *H2* and *H3*. Similarly, children’ self-concept of ability and interest serially mediated the relationship between parents’ sports-related behaviors and children’s sports activities (*β* = 0.009, *Z* = 1.800, *p* = 0.035), which supported *H4*. Furthermore, results from 5,000 bootstrap samples showed that the confidence intervals of the three indirect effects did not contain zero, thus validating the existence of these mediating effects. Finally, by comparing these three specific indirect effects, we found that children’ interest may be more critical than the other two factors (see Table 4).

**Table 4:** Direct, indirect, and total effects of the statistical model.

Relationships	Point estimation	Product of coefficients			Bootstrapping			
					BC 95% CI		Percentile 95% CI	
		SE	Z	*p*-value	Lower	Upper	Lower	Upper
Specific indirect effects								
PB → SCA → IN→SA	0.009	0.005	1.800	0.035	0.003	0.022	0.002	0.020
PB → SCA → SA	0.034	0.014	2.429	0.007	0.013	0.068	0.011	0.064
PB → IN→SA	0.097	0.027	3.593	0.000	0.053	0.159	0.051	0.156
Total indirect effect	0.139	0.035	3.971	0.000	0.079	0.216	0.078	0.213
Direct effect	0.326	0.048	6.792	0.000	0.235	0.426	0.233	0.423
Total effect	0.466	0.052	8.962	0.000	0.362	0.566	0.362	0.565
Contrasts								
SCA & IN vs. SCA	−0.025	0.013	−1.923	0.027	−0.058	−0.004	−0.055	−0.002
SCA & IN vs. IN	−0.088	0.025	−3.520	0.000	−0.149	−0.048	−0.144	−0.045
SCA vs. IN	−0.063	0.028	−2.250	0.012	−0.124	−0.014	−0.123	−0.014

*N = 367. 5,000 bootstrap sample; SE, standard error; BC, bias corrected; CI, confidence interval; PB, parents’ sports-related socialization behaviors; SCA, self-concept of ability; IN, interest; SA, sports activities.*

### 3.4 Multi-group model analysis by gender

To assess the applicability of the model across different genders, a multigroup analysis of structural invariance was conducted using structural equation modeling (see Table 5). First, two groups, boys (*M*_1_) and girls (*M*_2_), were set up in the structural equation model for group comparison. The results showed that, compared with the baseline model (*M*_3_), the measurement model (*M*_4_) did not exhibit a deterioration in fit after adding constraints (Δ*χ*^2^ = 8.812, Δ*df* = 10, *p* > 0.05), indicating that the measurement weights were invariant between boys and girls. Moreover, compared with Model 4, the structural model (M_5_) also did not show a deterioration in fit after adding constraints (Δ*χ*^2^ = 9.800, Δ*df* = 6, *p* > 0.05), suggesting that the structural weights were invariant between boys and girls. Taken together, the serial mediational model, in which parents’ sports-related socialization behaviors influence their children’s participation in organized after-school sports activities through the children’s ability beliefs and interests, is applicable to both boys and girls without significant gender differences (*H5*).

**Table 5:** Goodness-of-fit statistics and model comparisons for multisample structural models (*N* = 367).

Model	χ^2^	*df*	TLI	CFI	RMSEA	Δχ^2^	Δ*df*	*p*-vale
M_1_ Boys	95.099	71	0.982	0.986	0.044			
M_2_ Girls	95.988	71	0.982	0.986	0.043			
M_3_ Unconstrained baseline model	171.326	142	0.989	0.992	0.024			
M_4_ Measurement weights	180.138	152	0.990	0.992	0.023	8.812	10	0.550
M_5_ Structural weights	189.938	158	0.989	0.991	0.024	9.800	6	0.133

## 4 Discussion

The purpose of this study was to investigate the relationship between parents’ sports-related socialization behaviors and children’s participation in sports activities, with a focus on the mediating roles of self-concept of ability and interest. Guided by the framework of Eccles and Wigfield (2024) expectancy-value model, the findings reveal a complex interplay of direct and indirect pathways, offering new insights into the mechanisms that drive children’s sports activities engagement. The sample size of this study was 367, which exceeded the minimum sample size requirement and met the standard of the rule of thumb. The result of the statistical power test for SEM was 0.989, giving enough confidence in the correctness of the findings of this study (Kline, 2015; MacCallum et al., 1996). In addition, consistent with the model of parent socialization and relevant empirical studies, this study shows the existence of gender stereotypes in the field of children’s sports activities. For example, studies have found that parents provide significantly higher levels of material support to boys than to girls, a difference that likely reflects unconscious gender stereotypes held by parents (Hong et al., 2020; Jaf et al., 2021; Simpkins et al., 2012). However, despite the differences at the mean level, model analysis of different gender groups revealed that there were no significant differences in the serial mediation model of parental sports-related socialization behavior influencing children’s sports activities participation through children’s self-concept of ability and interests. Overall, this study provides additional empirical evidence for the broad applicability of parental socialization models among both boys and girls. Below, these findings are discussed in relation to existing research and their theoretical and practical implications are explored.

The first objective of this study was to investigate the relationship between parents’ sports-related socialization behaviors and children’s sports activities participation. The findings indicated that parents’ behaviors, such as encouragement, co-activity, provision of activity-related materials, and role-modeling, have a significant positive direct effect on children’s sports activities participation (*β* = 0.326, *Z* = 6.792, *p* < 0.001). This result aligns with prior research emphasizing the role of parents in shaping children’s sports activities habits (Liszewska et al., 2018; Reimers et al., 2019a). Specifically, the results highlight that parental encouragement and co-activity play pivotal roles in fostering children’s motivation and persistence in sports activities. These findings can be interpreted through Eccles and Wigfield (2024) motivational socialization model, which posits that parents act as key facilitators of children’s sports activities by providing an enabling environment and positive reinforcement. Moreover, parents’ provision of materials and role-modeling behavior are critical dimensions of their socialization efforts. For instance, previous research emphasizes that role modeling serves as a powerful mechanism for transferring values and fostering engagement (Carayanni et al., 2020; Wilk et al., 2018). Similarly, the provision of activity-related resources, though less examined, may address structural barriers to participation, such as access to equipment or transportation (Simpkins et al., 2015). While this study only investigated the overall dimensions of parental behavior, SEM analysis also seems to offer potential for these explanations, and future research can further investigate the unique contributions of each dimension. Taken together, the finding highlight the influence of parents in promoting children’s sports activities.

The second objective of this study was to examine the mediating role of children’s self-concept of ability in the relationship between parents’ socialization behaviors and children’s sports activities participation. The results revealed a significant indirect effect (*β* = 0.034, *Z* = 2.429, *p* = 0.007). These findings are consistent with the literature suggesting that the self-concept of ability serves as a critical mediator in the motivational process (Babkes and Weiss, 1999; Heitzler et al., 2010). For instance, when parents provide encouragement and act as positive role models, children are more likely to perceive themselves as competent, which, in turn, enhances their participation in sports activities (Jaf et al., 2021; Timperio et al., 2013). However, we cannot rule out the possibility of gender differences. For example, previous studies have elaborated on the gender stereotypes in sports activities, and that self-efficacy may be more closely associated with boys’ sports activities (Li and Moosbrugger, 2021). Conversely, one study has found that individuals’ beliefs about ability and value do not differ by gender (Simpkins et al., 2012). Considering that few studies have investigated gender as a moderating factor, it is necessary to replicate the research findings. Overall, this pathway highlights the importance of parental behaviors that nurture children’s confidence and competence in sports activities.

The third objective focused on the mediating role of children’s interest in the relationship between parental socialization behaviors and children’s sports activities participation. The findings demonstrated that interest significantly mediated this relationship (*β* = 0.097, *Z* = 3.593, *p* < 0.001). These results resonate with existing research, which indicates that parents who cultivate a supportive and enjoyable sports activities environment have a positive impact on children’s intrinsic motivation (Eccles et al., 2005; Eccles and Wigfield, 2024). For example, parental involvement in co-activities can create a positive and engaging atmosphere, enhancing children’s perception of the enjoyment and intrinsic value of sports activities (Pelletier et al., 2020). Such an approach is particularly critical, as interest has been shown to predict sustained participation in various contexts (Eccles and Wigfield, 2024; Viljaranta et al., 2014). However, these results must be interpreted with caution. Given the study’s focus on children, its findings may not be directly applicable to other age groups, and individual differences among children, such as cultural background and personal experiences (Jaf et al., 2021), may modulate the observed relationships.

Finally, this study investigated the serial mediation effect of self-concept of ability and interest in the relationship between parents’ sports-related socialization behaviors and children’s after-school organized sports activities participation. The findings supported that self-concept of ability and interest jointly mediate this relationship (*β* = 0.009, *Z* = 1.800, *p* = 0.035). This indicates that parents’ supportive behaviors can shape children’s self-concept of ability, which in turn stimulates their intrinsic interest, thus promoting their participation in sports activities. Although this result has not been reported in previous research on sports activities, by integrating the studies of Pelletier et al. (2020), Simpkins et al. (2012), and Viljaranta et al. (2014), it can be inferred that there is a serial mediating effect of ability beliefs and interest between parental behaviors and children’s sports activities. One possible explanation is that individuals’ positive perception of their own abilities (i.e., self-concept of ability) enhances their expectations of success in an activity, which in turn makes them perceive the activity as more valuable, thus triggering and intensifying their interest in the activity (Eccles and Wigfield, 2024). Taken together, these results highlight the critical role of parents in promoting children’s sports activities through direct support and by fostering positive psychological mechanisms. While the current study identifies interest as a potentially more significant mediator, both mediating pathways contribute meaningfully to the overall process. Therefore, interventions targeting parental support should emphasize the cultivation of children’s self-concept of ability and interest to maximize their engagement in sports activities.

### 4.1 Contributions and implications

This study provides valuable insights into the mechanisms by which parents’ sports-related socialization behaviors influence children’s sports activities participation. By highlighting the direct and indirect effects of parental socialization behaviors, this research contributes to the literature on youth sports activities. The findings emphasize that creating an environment where parents actively participate, encourage, and provide resource support for sports activities is crucial for enhancing children’s participation in sports activities. For practitioners and policymakers, this study suggests that interventions aimed at increasing youth sports activities should incorporate strategies to educate parents about effective socialization behaviors. For example, through parent workshops or community projects, parents can be guided on how to encourage, demonstrate, and promote children’s sports activities. Similarly, schools and sports organizations can collaborate with parents to create a coordinated support system that emphasizes the development of children’s self-concept of ability and interest (e.g., organized co-activities or family-based sports activities programs). Additionally, explore the potential of public health promotion campaigns in addressing the barriers to parental involvement (e.g., lack of time or resources) to promote parental support for adolescent sports activities.

### 4.2 Limitations and future research

Although the present study provided meaningful contributions, there were several limitations. First, the cross-sectional design of this study limited causal inferences. Future work could consider adopting a longitudinal design to explore the dynamic relationships between these variables over time. Second, the sample of this study was limited to urban children from one single region in China, which may limit the generalizability of the findings. In future studies, expanding the sample to include different regions and socio-economic backgrounds would strengthen the robustness of the results. A third limitation is that data collection used self-reports from children aged 10 to 12 years, which may have measurement biases and also limit generality (Lynn, 2000; Sallis and Saelens, 2000). Further research should incorporate objective measures and cover a broader age range. A fourth limitation is that the study did not adequately consider the potential differences between fathers and mothers. Future research should consider differences between fathers and mothers, as well as diverse family structure factors such as single-parent families. Moreover, exploring the role of cultural factors and individual differences in shaping these relationships would contribute to a more comprehensive understanding of how parental behaviors influence children’s sports activities participation. Overall, by addressing these limitations, future research could build on the current findings to develop more targeted and effective intervention strategies.

## 5 Conclusion

The present study was designed to determine the effect of parents’ sports-related socialization behaviors in influencing children’s after-school organized sports activities participation, with a particular focus on the mediating roles of self-concept of ability and interest. The findings revealed that parental socialization behaviors not only have a direct impact on children’s sports activities participation but also have an indirect influence through self-concept of ability and interest, as well as their serial mediating effects. The current data highlights the critical role of parents in shaping children’s sports activities habits and the importance of creating supportive environments to foster positive self-perceptions and intrinsic motivation. In conclusion, this study provides a nuanced understanding of the mechanisms linking parental support to children’s sports activities participation. By providing a multiple mediation model, the findings offer practical guidance for designing interventions aimed at promoting children’s sports activities. Future research should continue to explore these relationships, addressing the identified limitations and expanding the scope of investigation to provide more comprehensive insights into children’s sports activities motivation.

## Data Availability

The raw data supporting the conclusions of this article will be made available by the authors, without undue reservation.
